# The application of GPR to the detection of soil wetted bodies formed by drip irrigation

**DOI:** 10.1371/journal.pone.0235489

**Published:** 2020-07-22

**Authors:** Gao Peng, Wang Ruiyan, Zhao Gengxing, Li Yuhuan

**Affiliations:** 1 College of Resources and Environment, Shandong Agricultural University, Tai'an, China; 2 National Engineering Laboratory for Efficient Utilization of Soil and Fertilizer Resources, Tai'an, China; China University of Mining and Technology, CHINA

## Abstract

The ability to accurately measure the geometric characteristics of soil wetted bodies (SWBs) is very important for conserving water in agriculture. However, measurements of SWBs obtained using conventional methods have a number of defects. Ground penetrating radar (GPR) is a promising technique for detecting buried features. In this paper, we used GPR to nondestructively investigate SWBs formed under drip irrigation. First, numerical simulations were performed to obtain the theoretical radar-gram of SWBs. Then, controlled irrigation experiments were performed to obtain radar scan datasets in a laboratory. The GPR image was interpreted according to the numerical simulation results, and the SWB thickness detection accuracy was estimated. Finally, GPR detection was performed in the field with different irrigation volumes, and the detection effect was assessed. The GPR reflections in the laboratory and field measurements were more complex than in the numerical simulation images, but the location and thickness of SWBs were still clear; the accuracy of the measured thickness was high, and the accuracy decreased with an increase in irrigation volume. The radar image resolution and thickness accuracy measured in the field were slightly less than the values measured in the laboratory. Thus, GPR is able to quickly and accurately characterize SWBs formed by drip irrigation based on the thickness and relative position in the soil. Furthermore, the F-K offset transformation was an effective GPR data processing method for focusing reflections from SWBs to obtain their true position and physical extent.

## Introduction

Water resource scarcity in many regions of the world, combined with the large amount of water used in agriculture, is promoting the adoption of more efficient irrigation practices [1]. Developing effective irrigation and drainage strategies to improve the quality of soil is vital for enhancing agricultural production and increasing economic returns [2]. The efficiency of irrigation and water use in China is only 52%, which is far lower than the 70–80% efficiency in developed countries. To reduce water shortages, it is urgent to reduce water irrigation [3]. In China, water shortages are important factors restricting agricultural development. Improved water-saving agricultural practices are needed for China to exploit the country’s natural conditions and promote agricultural development [4, 5]. Drip irrigation is prominent among the proposed solutions to the water crisis because this method conserves water, increases crop production, and improves crop quality [6, 7]; therefore, drip irrigation techniques are popular water-saving agriculture applications [8, 9]. In comparison with flood irrigation and furrow irrigation, drip irrigation has the obvious advantage of high irrigation efficiency [10]. Drip irrigation technology has broad application prospects, particularly in arid areas, such as in western China [11].

The rapid, high-precision and non-destructive determination of shallow soil water content (SWC) is of vital importance to precision agriculture and water resource management [12]. The soil wetted body (SWB) is the moist area of soil that occurs under partial irrigation conditions. The establishment of an efficient irrigation system and acquisition of precise irrigation are important for optimal crop growth. Consequently, knowledge of characteristics such as the location, size and shape of the SWB must be obtained [1]. Various techniques are currently being used to measure these morphological parameters of SWBs in situ [13]. The most reliable method is excavating a soil pit to measure the extent and depth of SWB. However, this method is time-consuming, costly and invasive, and the ability to visually distinguish the wetted soils from the dry soils is sometimes difficult. Additionally, because SWBs are spatially variable, this measurement technique is of limited value when used over large areas. For these reasons, this technique is rarely used [8]. In comparison, an alternative method is the use of numerical, empirical or physical hydraulic models, such as the Schwartz mass and Zur and Richards’ equations. These models have been used to obtain the width and depth of the wetted soil zone. However, due to the substantial variation in the hydraulic properties of soils, these predictions are not always reliable [14]. Another method is the use of transparent rings in filtrometers made of Plexiglas to observe and measure the soil moisture distribution formed by emitters placed on the corner of the device in laboratory experiments [15]. Using this method, the SWB boundary is visualized during water flow. However, the preferential flows along the ring walls are not consistent with the wetted bodies formed in the natural state. Therefore, alternative techniques that allow SWBs to be detected in situ and nondestructively are in high demand. In this context, the use of ground penetrating radar (GPR) provides new perspectives for SWB measurements in situ.

GPR is a detection technology based on the reflection of electromagnetic waves radiated from a transmitting antenna. The distribution of different materials within the medium is determined by the reflection of these waves as they travel across an electromagnetic contrast or boundary [16, 17]. One of the most promising geophysical methods used to measure SWC is ground penetrating radar (GPR) because of the high sensitivity of the GPR wave velocity to changes in SWC [18].The GPR method is nonintrusive and has a large sampling volume; these features are advantageous in investigations of soil properties at the field scale [9, 19], such as studies of the detection of soil water, roots, groundwater tables, and water leaks from buried pipes [20–29].

Bano and Loeffler [30] used a 1200 MHz antenna to simulate two levels of the water table (at 72 and 48 cm depths) by injecting water into a sand box that also contained buried objects, and the researchers analyzed the reasons why the GPR profiles did not show any clear reflections from the top of the phreatic surface. Mahmoudzadeh and colleagues applied GPR with a single 200 MHz bowtie antenna in a semiarid catchment (Sardon, Salamanca, Spain) to investigate the water table depth in weathered granites [31]. Numerous studies have used GPR to detect the base of the melted active layer of the permafrost in summer [32]. An experiment by Demirci and colleagues confirmed the ability of GPR to accurately locate sources of leaks [4]. Most of these studies used GPR to detect the phreatic surface and water saturation zone. However, at present, few studies have used GPR techniques to investigate SWBs formed by drip irrigation.

The moisture content inside an SWB is much higher than the surrounding soil, and this contrast between the external and internal zones of the SWB results in a significant change in dielectric permittivity, which creates strong electromagnetic wave reflections. This boundary forms a reflective interface with GPR. Therefore, we propose that GPR is useful for obtaining subsurface distributions of SWB under drip irrigation. In this paper, the SWB of drip irrigation is used as the study object and the synthetic GPR data are modeled to analyze the GPR responses of SWBs with different sizes. Based on these results, real data measured in laboratory dry soil and in field soil are interpreted.

The primary objectives of this study were to (1) determine the particular features occurring in SWB radar images; (2) develop GPR radar-gram interpretation techniques for obtaining reasonable and rapid estimations of SWB; and (3) investigate the applicability of GPR for detecting wetted bodies with much greater resolution than can be obtained with conventional measurements. This research provides a new technique for detecting SWBs under drip irrigation.

## Materials and methods

### Experimental materials

#### Preparing the experimental soil

This study was carried out in the experimental site of Shandong Agricultural University. The soil was collected from the local cultivated land. The experimental soil was obtained without contamination from the topsoil to a depth of 0.2 m in the field and was dried naturally. The laboratory analysis of the experimental soil indicated that the average bulk density is 1.32 g/m^3^, and the initial volumetric moisture content is 0.02. Then, we sieved the soil through a 2.0 mm sieve.

Using the United States Department of Agriculture (USDA) classification for soil separates (USDA Soil Division Staff, 2017), an analysis of a sample from the experimental soil showed that 3% of the particles have a diameter of less than 0.002 mm, and 53% of the particle sizes range from 0.002–0.05 mm, as shown in Table 1.

**Table 1:** Soil texture and bulk density of the experimental soil.

Texture class	Soil particle size distribution			Soil bulk density
	Sand (2–0.05 mm)	Silt (0.05–0.002 mm)	Clay (<0.002 mm)	
Silt (sand) loam	44%	53%	3%	1.32 g/m^3^

These values are typical for silt loam.

#### Preparing the experimental box

**Fig 1:**
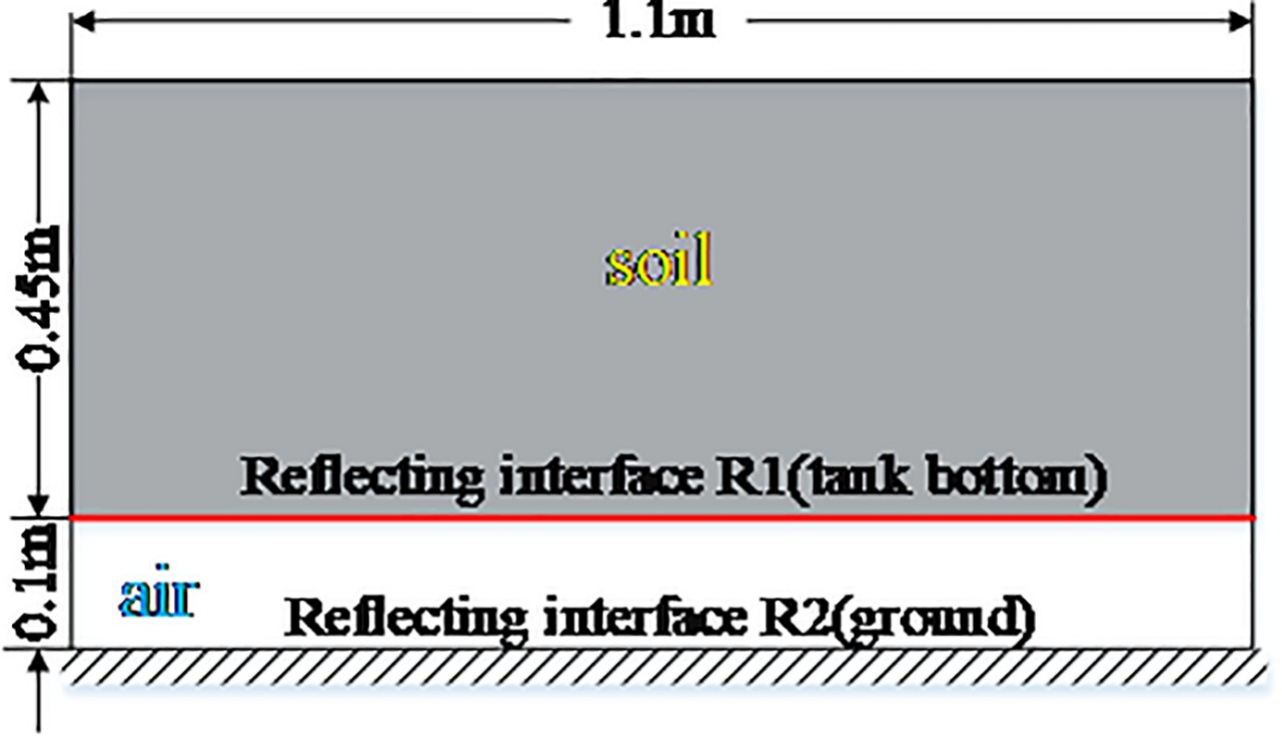
Schematic of the experimental box used for the SWB irrigation test.

An experimental box for the SWB irrigation test was constructed out of wood with dimensions of 1.1×0.6×0.45 m (L × W × D) (see Fig 1). The box had an open top and four legs, one on each corner of the box bottom, which placed the box 0.1 m above the ground.

### Acquisition and processing of GPR data

Because the simulated SWB is small, a high-frequency GPR antenna was used to obtain high-resolution images of the SWB characteristics. Therefore, all GPR measurements were performed using an LTD-2100 system (manufactured by Institute of Radio Communication, China) with a shielded 1.5 GHz antenna that was configured in the common fixed offset mode (the distance between GPR transmitter antenna (T_X_) and receiver antenna (Rx) is fixed). The 1.5 GHz antenna was placed on the soil surface within the experimental box and moved across the SWB without touching the side walls of the box. In addition, because the electromagnetic field increased significantly when the electromagnetic wave traveled in the SWB, the clipping wave phenomenon was significant. Clipping is caused by inappropriate (excessively high) range gain settings. The amplitude of the reflected signal increases with contact with more highly contrasting features. A segment gain algorithm was used to adjust the radar gain parameters. In addition, as the time window on the LTD-2100 system determines the profiling depth scanned on the radar profile, the time window was set to 9 ns (nanoseconds), and the sampling interval was set to 0.11 ns. When these parameters were set, the signal from the lower SWB position was strengthened, and the strong reflection from the upper SWB position was weakened.

We used the radar data processing software IDS 6.0 to process the data. Common post-processing steps applied to the original radar data included zero offset, gain, filter, clipping and stitching processes.

### Determination of the soil volumetric moisture content

After the GPR data scan was performed for each irrigated volume, the SWB was extracted from the box, and the wetted body was divided into three layers according to thickness after excavation. Three soil samples were collected from each layer to measure the soil moisture content. Soil bulk density was measured using the ring knife method. These samples were used to determine the moisture content with the oven drying method. Finally, the soil volumetric moisture content of the soil samples was calculated from the bulk density and moisture content.

### Estimation of SWB thickness and dielectric constants

#### Estimation of the dielectric constants

The GPR numerical simulation requires the dielectric constant inputs for the SWB and dry experimental soil. Considering the GPR scan mode mentioned above, dielectric constants for the media were estimated using the wave velocity method. The radar wave velocity is mainly affected by the dielectric constant of the scanned medium, and the soil dielectric constant *ε_r_*, which can be approximately calculated using Eq (1):

$$\varepsilon_{r}={\left(c/v\right)}^{2}$$ (1)

where *c* is the velocity of light in a vacuum (0.3 m/ns), *ε_r_* is the relative permittivity of the scanned medium, and *v* is the radar wave velocity. In dry soil, *v* is represented by *v_S_*, and in the SWB, *v* is represented by *v_W_*.

#### Wave velocity estimation

When the experimental box was filled with dry soil, the GPR was moved across the soil surface to capture a radar-gram. The two-way pulse travel time to the reflection produced by the bottom of the wooden box and the thickness of the dry soil were measured and used to estimate the radar wave velocity, *v_S_*. In the experiment, the distance is close between the two antennas, and so the antenna spacing is ignored, as shown in Eq (2):

$$v_{S}=2h_{b}/t_{b}$$ (2)

where *h*_b_ is the dry soil thickness in the box (*m*) and *t*_b_ is the two-way travel time of the wave through the dry soil column (ns).

The moisture was assumed to be approximately uniformly distributed inside the SWB, and the average volumetric moisture content was similar for each SWB. Accordingly, the radar wave velocity was assumed to be the same in each of the SWBs. In our experiment, the estimated radar wave velocity inside the SWB of a 200 ml irrigation volume was used for all SWBs. The following formula was used to calculate the radar wave velocity:

$$v_{w}=2h_{2}/t_{2}$$ (3)

where *h*_2_ is the SWB thickness of the 200 ml irrigation volume (*m*) and *t*_2_ is the two-way travel time (ns) of the wave inside the SWB of the 200 ml irrigation volume.

#### Estimation of the SWB thickness

The SWB thickness was estimated using the radar velocity *v_w_* and the radar wave travel time inside the SWB with Eq (4):

$$h=0.5v_{w}t$$ (4)

where *h* is the SWB thickness of the 200 ml irrigation volume (*m*) and *t* is the two-way travel time (ns) of the wave inside the SWB of the 200 ml irrigation volume.

### GPR numerical simulation of SWBs with different irrigation volumes

**Fig 2:**
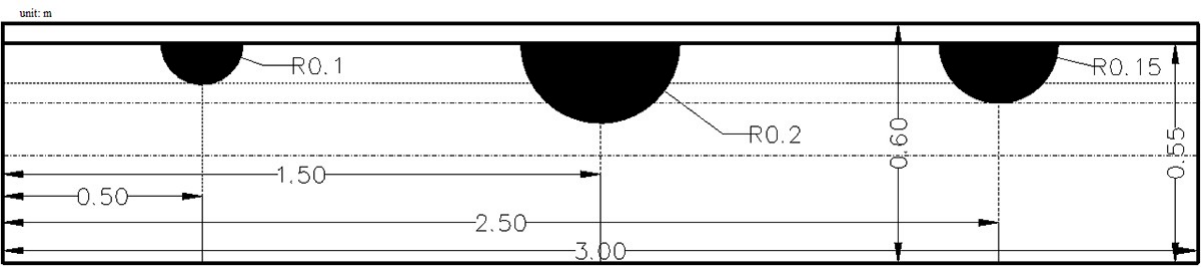
GPR forward simulation model of the configuration of SWBs with different irrigation volumes.

For the purpose of consistency with the GPR radar-grams in the subsequent survey experiments, we simulated the radar echo signal from the soil profile just below the dripper. According to previous studies, in a homogenous and unlayered medium, the SWB presents a shape similar to a hemisphere when using surface drip irrigation [6]. Irrigation volumes used in the numerical simulation experiment were 200 ml, 400 ml and 600 ml. The thicknesses of these simulated SWBs formed 1 m gap serials to highlight the GPR profiles of the different SWB sizes and were larger than the measured thickness. As shown in the sketch of the simulation model (Fig 2), the center coordinates of the three wetted bodies were spaced at horizontal distances of 0.5, 1.5, and 2.5 m.

The simulated soil parameters were referenced to the experimental soil described above to increase the validity of the numerical simulation. Conductivity affects the propagation of electromagnetic waves in the medium. The soil conductivity is mainly dominated by the moisture content, and an exponential correlation exists between these parameters (R^2^ = 0.71) [13]. The soil conductivity was calculated as 0.0057 (S/m), and using the same method, the SWB conductivity was 0.018 (s/m). The dielectric constants were calculated from formulas (1) and (2), and the dielectric constants of the SWB and dry soil were 14.51 and 3.12, respectively. The dielectric constant of air was 1, and the air conductivity was 0. Most of the soil was non-depleted magnetic media; therefore, the soil and SWB permeability were set to 1.

In addition, the mesh was set to dimensions of 2.5 mm by 2.5 mm in a domain of 3 m by 0.6 m. The PML (perfectly matched layer) absorption layer number was 10, the measuring point spacing was 0.01 m, the scan line length was 2.89 m, the antenna spacing was 0.025 m, and the air layer thickness was 0.05 m.

According to the parameters and configuration of the model described above and using the program compiled by MATLAB and GprMax2D based on the finite-difference time-domain (FDTD) method, the simulation experiment for radar waves of SWBs under three drip irrigation volumes was performed.

### Approximation of the real shape of the SWB

The observed shapes of the SWBs were different from those in the radar-grams, and thus the radar-grams were difficult to interpret. The F-K offset algorithm (frequency-wave domain migration algorithm) transforms the wave equation in the time-space domain into the frequency-wavenumber domain to achieve offset homing. In this paper, this algorithm was used to approximate the real shape of the SWB from the GPR image.

In the present study, we used REFLEX-Win 5.0 software to process the GPR radar-grams with F-K migration. The preprocessed radar-grams were imported into this software, and the filtering parameters were set according to the data attributes. The attributes included the conversion coefficient, wave velocity and starting time. The conversion coefficient was set to 3 and the wave velocity was estimated. After setting these parameters, the migration process was performed.

### Laboratory measurement of SWBs

**Fig 3:**
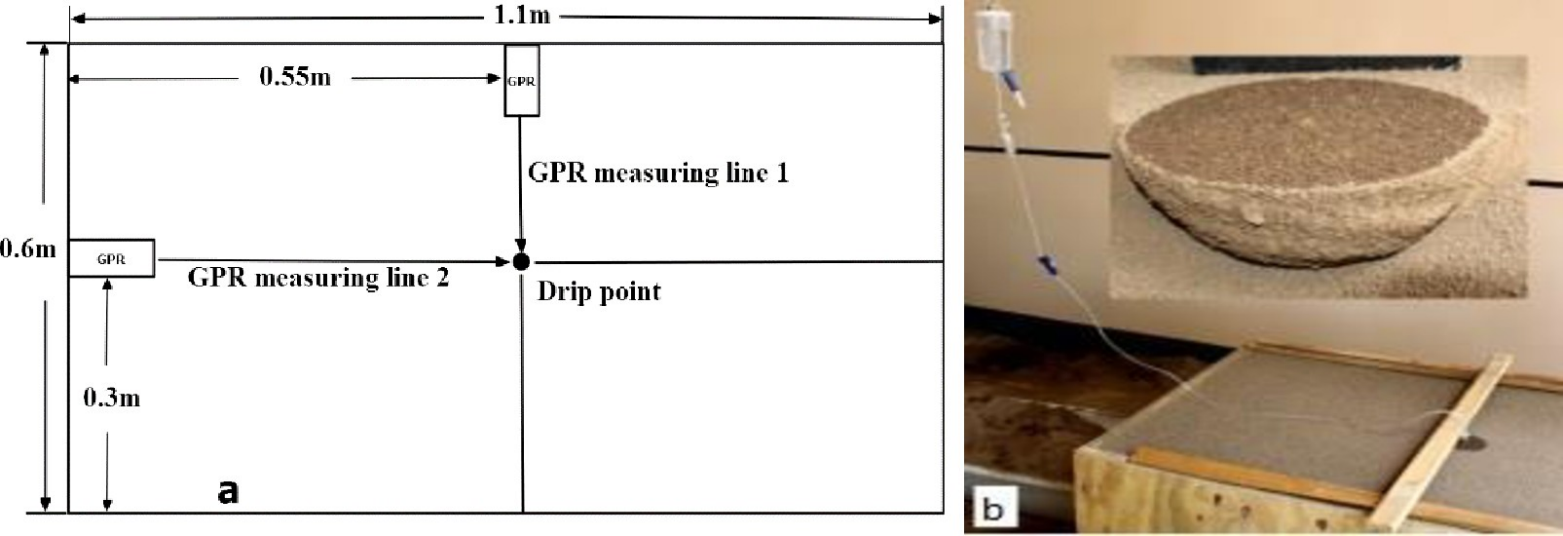
Images of the real measurement configuration and excavation of the SWBs.

Consistent with the numerical simulation, the irrigation volumes of the three SWBs were designed to be 200 ml, 400 ml and 600 ml. The experiment was performed in the wooden experiment box. The wooden box was filled with experimental soil and smoothed. In the drip irrigation experiment, the catheter of a drip PVC infusion device was placed at a height of 0.5 cm above the soil surface in the center of the experimental box (Fig 3A) and emitted water at a flow rate of 4 ml/min. A photograph of the irrigation experiment is presented in Fig 3B. Two hours after the irrigation water was applied, when the soil moisture was considered no longer spreading and the GPR scan began.

The GPR scan was conducted in the test box along line 1 and line 2 (see Fig 3A). GPR scanning data from the SWBs with different irrigation levels were obtained. When GPR scanning was completed, we manually excavated the soil to ensure the integrity of SWB. After the excavation was completed, its size was measured, and a circular knife was used to retrieve soil samples and determine the average volume moisture content of the SWB.

### In situ GPR measurement of SWBs in the field

The main purpose of this experiment was to investigate the feasibility of using GPR to detect and image SWBs in the field. The experimental site was located in Shandong Agricultural University farm in a field that had not been planted or cultivated, where we obtained the laboratory soil. The undisturbed soil in the field had the same texture as the laboratory soil but had not been treated. However, due to recent rain, the subsurface soil moisture content in the field was higher. Assuming that the irrigation volume was usually greater in practice than in the laboratory experiment, five irrigation volumes were used: 500 ml, 1000 ml, 1500 ml, 2000 ml, and 2500 ml.

**Fig 4:**
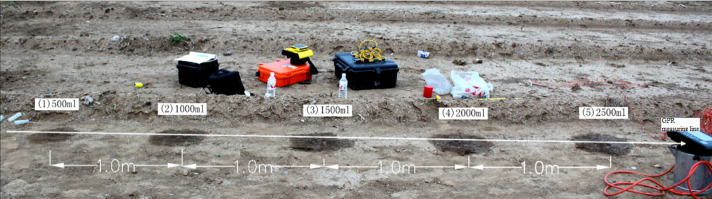
Sketch map of the field experiment.

In the field experiment, a line of irrigation points spaced at 1 m intervals was established (Fig 4). The GPR scan began two hours after the application of irrigation water ended. The approximate location of the GPR scan line at the field site is shown in Fig 4 (white lines). The collected radar-gram is consistent with the principle of using a 200 ml SWB to obtain the wave velocity in the laboratory experiment. The radar wave velocity that was calculated inside the SWB of the 500 ml irrigation volume was used to determine the thicknesses of all SWBs. The same radar data processing methods were used for the radar-grams collected at the field site as in the laboratory experiment.

### Accuracy of GPR at detecting the SWB thickness

On each of the original GPR radar-grams, the two-way travel time from the surface to the lower interface of an SWB was measured, and then formulas (3) and (4) were combined to calculate the SWB thickness. The calculated SWB thickness was compared with the actual measured value to determine the GPR detection error for the SWB thickness using formula (5):

$$\Delta H=(FW_{a}-{FW}_{b})/{FW}_{b}*100$$ (5)

where Δ*H* is the error in the GPR-detected thickness, *FW*_a_ is the GPR-detected thickness, and *FW*_b_ is the measured thickness.

## Results

### Results from the numerical simulation and analysis

**Fig 5:**
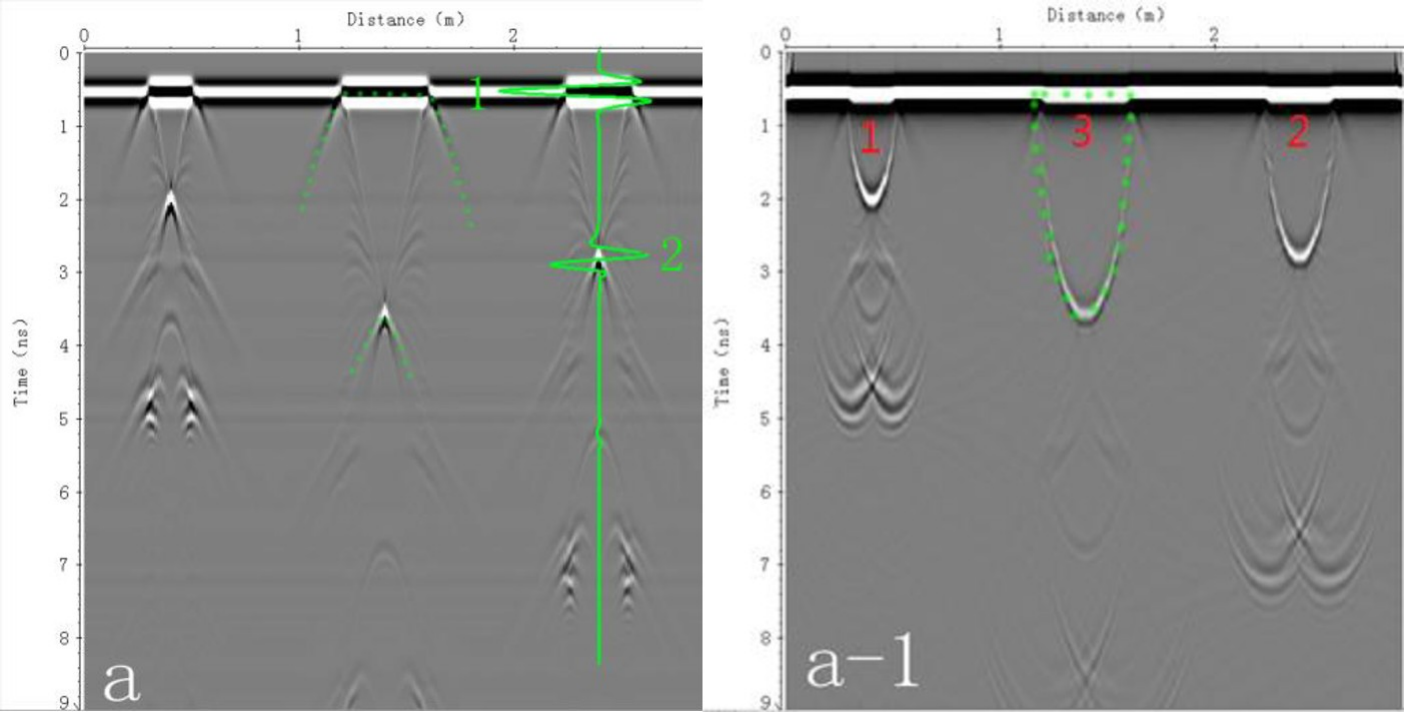
A original graph; A-1 results of F-K migration processing.

The radar image obtained from the numerical simulation model and the F-K migration transformation result of the image are shown in Fig 5A and 5A-1, respectively, In Fig 5A, three SWB reflection patterns appear on the modeled radar response. These reflection patterns appear as high-amplitude hyperbolas (caused by a wide antenna radiation pattern) composed of alternating bands of positive and negative polarity. Their high reflection amplitudes are due to the significant contrast in dielectric permittivity between the external and internal zones of the SWB and the small signal losses, which makes these features identifiable. Both surface and subsurface reflections are evident for the three applied irrigation volumes (200, 400 and 600 ml) in Fig 5A. A green-colored vertical line has been used to identify the center of the 600 ml irrigation application point. The top and bottom interfaces of the SWB are represented by two reflection curves that have been highlighted by horizontal green lines. The upper interface of the SWB appears at a 2-way travel time of 0.5 ns (see vertical scale) as a straight line with half curves at each end.

In Fig 5A-1, labels 1, 2, and 3 represent the irrigation volumes of 200, 400, and 600 ml, respectively.

The two half curves mark the boundaries and define the length of the SWB surface diameter. For example, in Fig 5A-1, the wet body is marked as 2 with a 2-way travel time of approximately 2.8 ns, and the bottom of the SWB is represented as a downward opening hyperbola.

In addition, the un-migrated synthetic radar-gram is essentially free of clutter other than reflection banding and reflection multiples or reverberations. The absence of clutter is attributed to the numerical simulated soil model representing an ideal medium with a single texture and uniform composition. Multiple reflections are obvious under the SWBs, and the banded reflection waveforms of these multiples are the same shape as the initial reflections. These phenomena are caused by the reflection of the transmitted waveform back and forth between the surface and the reflecting feature and the GPR signal polarity. As the irrigation volume increases, multiple waves gradually weaken and the echo wave position moves downward, because a greater irrigation volume results in a thicker SWB and greater travel time and attenuation of the electromagnetic wave.

The F-K offset image shows that the hyperbola and radar wave energy of the SWB simulated data are converged, gathered and appear as upwardly opening hyperbolas on the image, which is the opposite of their shape in the original image.

### Results and analysis of laboratory measurements

#### Measured results and analysis of GPR data

**Fig 6:**
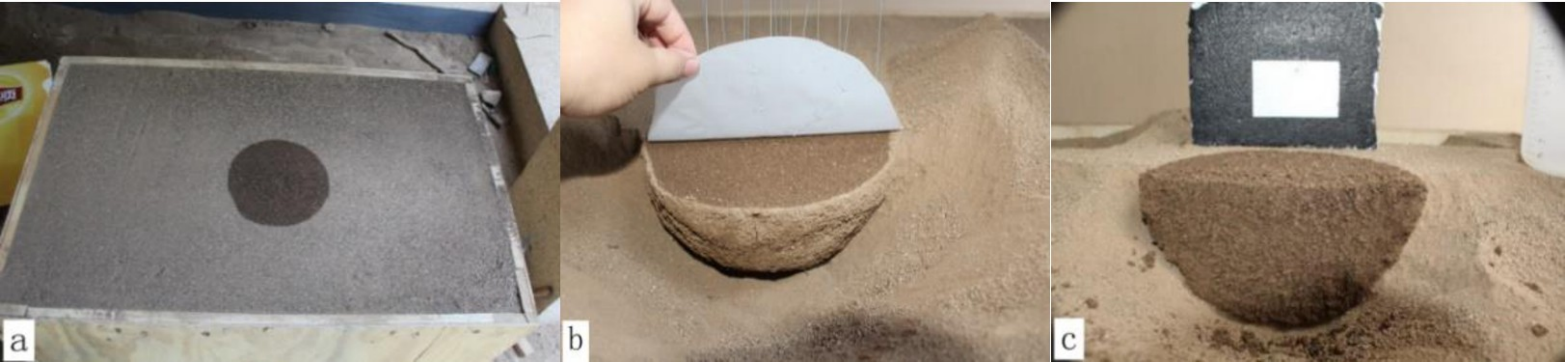
Results of SWB excavation after drip irrigation.

**Fig 7:**
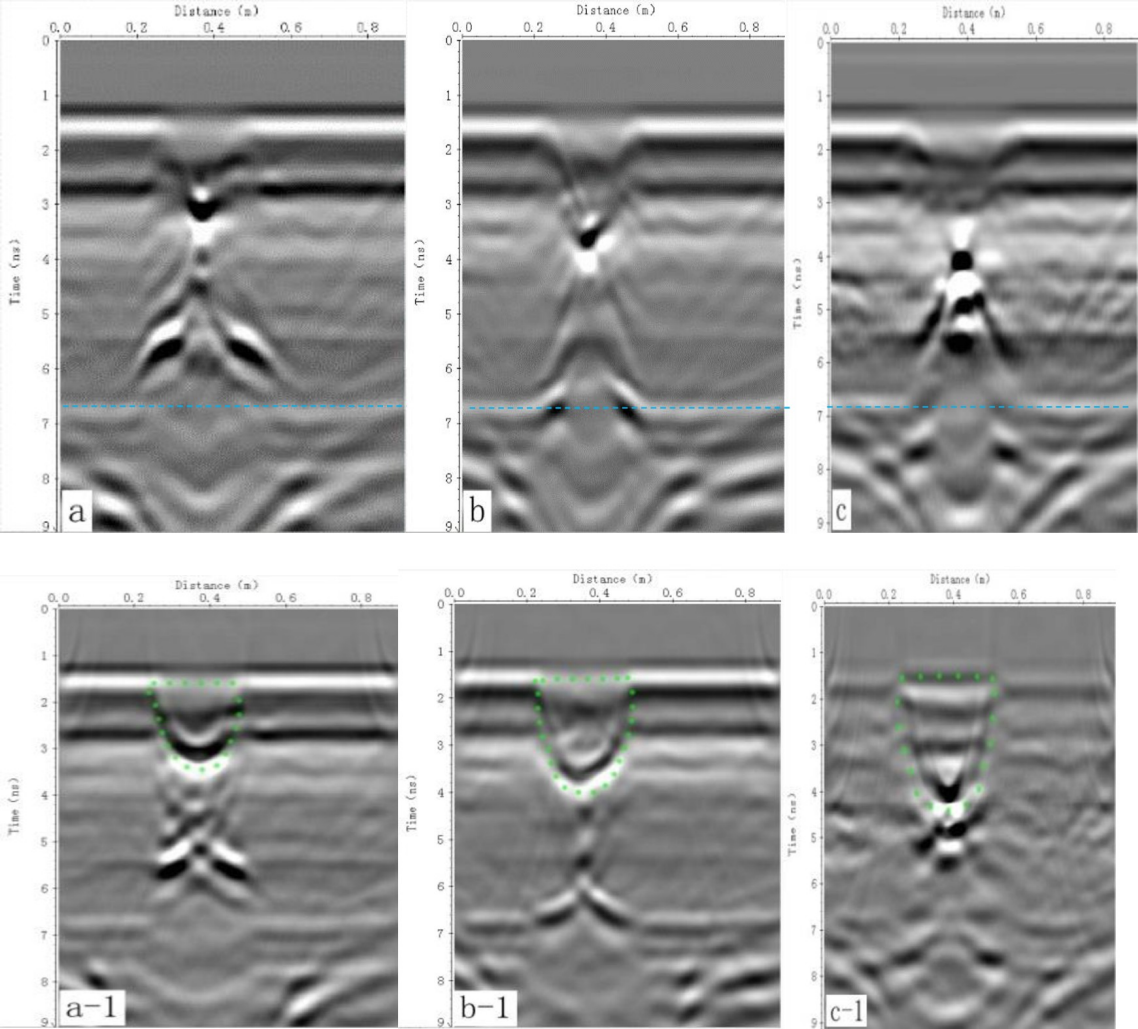
The original radar-gram and its treatment. Section maps A, B and C show original radar-grams for the 200 ml, 400 ml and 600 ml irrigation volumes, respectively; section maps A-1, B-1 and C-1 show F-K offset transformation results corresponding to maps A, B and C, respectively.

The upper surface and excavated profile of the SWB after drip irrigation are shown in Fig 6. The SWB showed a regular circular shape on the soil surface (Fig 6A). The whole body showed a hemispherical shape (Fig 6B), and the middle, vertically sliced profile showed a semicircular shape (Fig 6C). Sections of the original radar-gram and its F-K offset transformation for different irrigation volumes are shown in Fig 7A-1 and 7C-1.

In the original GPR radar-grams (Fig 7A–7C), the SWBs are clearly discernible, and the image characteristics are similar to the numerical simulation. The two edges of the upper surface are a half curve; the bottom interface is still an open hyperbolic curve. However, the upper surface of the SWB appears to be blurred and exhibit a slightly concave shape facing upwards rather than horizontal shapes, and the line width of the upper interface increases. These results are consistent with the results of the numerical simulation. The results of the SWB numerical simulation are helpful for understanding the GPR image features, which provide an effective reference and guidance for interpretation of the GPR-measured results.

However, the images of the un-migrated GPR radar-grams from the experimental box (Fig 7A–7C) are more complicated than the images from the numerical simulation (Fig 5).

In addition to reflections from the SWB, other wave reflections and clutter appear in Fig 7. Clutter caused by the unwanted reflections from the sidewalls of the wooden box are observed below the reflections from the surface and the SWB that were intercepted in the concealed radiation pattern of the antenna, reflections from soil layers, and reflections from the box bottom (highlighted by dashed blue lines in Fig 7A and 7B).

The F-K offset transformation radar-grams (Fig 7A-1–7A-3) show that the hyperbolas of the SWB are converged and gathered, which further improves their horizontal resolution. In the F-K offset transformation images, the SWBs show a nearly semicircular shape, similar to the shape of the excavation profile. From the migrated radar-grams of different irrigation volumes (Fig 7A-1–7C-1), the thickness of the SWB appears to increase with the irrigation volume. The SWBs resemble downwardly elongated spindles on these migrated radar-grams, similar to the shape in the numerical simulation model; therefore, the F-K offset transformation of the GPR radar-gram reveals the real shape, size, boundary and location of an SWB.

#### Accuracy of the thickness detection

The accuracy of the GPR-detected SWB thicknesses are shown in Table 2.

**Table 2:** GPR detection errors in the SWB thicknesses in the laboratory experiment.

Irrigation volume (ml)	Measured value (cm)	GPR detection value (cm)	Thickness error
200	8.2	8.2	-
400	10.3	10.93	6.15%
600	12.22	13.21	8.11%

Overall, Table 2 shows an average SWB thickness error of 7.13%, indicating that the detection of SWBs with GPR is a feasible and effective method and can be used in practical applications. However, as indicated in Table 2, the thickness measured using this method is slightly greater than the real value, and a larger error is obtained with an increase in irrigation volume.

### Field measurement results and analysis

#### GPR profile of the characteristics of the SWB

**Fig 8:**
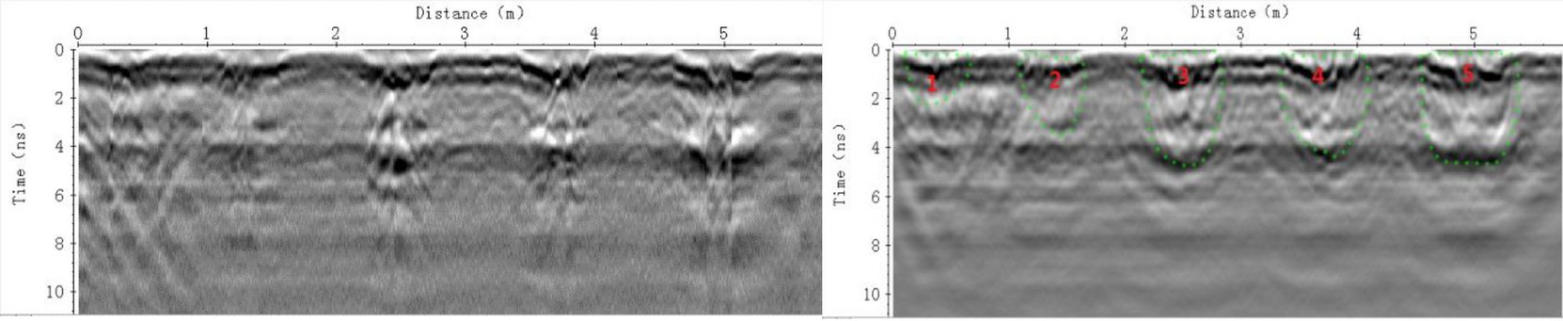
Original GPR radar-gram and the F-K migration processed image in the field.

The top and bottom images in Fig 8 are the original, un-migrated GPR radar-gram and the F-K offset migrated radar-gram from the field site. The labeled annotations 1, 2, 3, 4 and 5 in the lower, F-K offset migrated radar-gram identify the drip locations where different irrigation volumes of 500, 1000, 1500, 2000 and 2500 ml, respectively, were applied. Based on the original, un-migrated GPR radar-gram, the soil appears to contain some unwanted clutter and to be weakly stratified (white dashed line). In this radar-gram, images of the SWBs are similar to the images obtained in the laboratory (Fig 7A and 7B), presumably because of similarities in soil and dielectric properties between SWBs and the surrounding soil. In the un-migrated radar-grams (Fig 8, upper panel), the SWBs show obvious radar wave perturbations that indicate the approximate SWB locations and thicknesses.

However, the un-migrated GPR radar-gram (Fig 8, upper panel) appears to contain more clutter than radar-grams from the laboratory experiments (Fig 7A–7C), and the upper and lower interface shapes of the SWBs appear more irregular.

These conditions are attributed to the greater uniformity and less spatial variability of the properties of the laboratory soil than the soil at the field site. Under natural field conditions, the movement of water in soil is more random due to soil heterogeneity and preferential flow. Furthermore, as the irrigation volume increased, the expanse of the SWB reflection hyperbolas on the radar-gram gradually increased. Notably, the waveforms of the wetted bodies corresponding to 500 ml and 1000 ml SWBs on this radar-gram (Fig 8, upper panel) are more difficult to identify due to their more restricted development and the effects of the strong surface reflection. In addition, the bottom interfaces of the SWBs for the 1500 ml, 2000 ml and 2500 ml irrigation volumes appear to occur at a uniform time interval (or depth), which suggests a restricting soil layer. This phenomenon implies that the soil water flow expands from the vertical to the horizontal direction with increasing irrigation volume. For clarification, we excavated the soil and found that the surface soil is soft due to tillage, while the soil below the SWB is hard and dense, which hinders water diffusion in the soil. The bottom image in Fig 8 shows the radar-gram from the field site after F-K offset migration, and the dashed green line indicates the interpreted SWB boundary. As evident on this processed radar-gram, the shape and range of the SWBs for the 1000 ml, 1500 ml, 2000 ml and 2500 ml irrigation volumes are easier to identify than for the 500 ml irrigation volume. Thus, GPR is more suitable for detecting larger SWBs due to the effects of environmental factors and soil media heterogeneity on the field survey, which is not consistent with the laboratory experiments.

#### Errors in the GPR-detected SWB thickness

The errors in the GPR-detected SWB thickness in the field were calculated using the same method as the laboratory experiments. The detection errors are shown in Table 3.

**Table 3:** GPR detection errors of the SWB thicknesses in the field.

Irrigation volume (ml)	Measured value (cm)	GPR detection value (cm)	Thickness error
500	15.10	15.10	-
1000	20.70	21.98	6.18%
1500	28.00	30.51	8.96%
2000	26.40	27.89	5.65%
2500	27.60	30.18	9.35%

In general, Table 3 shows a greater SWB thickness detected using GPR is than the measured data in the field experiment, and the mean error is 8%, which is considered accurate from a practical perspective and can meet the needs of drip irrigation management. However, this error is slightly larger than the error of the laboratory experiment. The explanation for this difference may be related to the differences between the field soil and laboratory soil; the experimental soil is uniform, but soil properties, such as the texture, density, and moisture content, vary on the field scale, resulting in differences in the wave velocity between SWBs. However, the relationship between the accuracy and SWB thickness is consistent with the laboratory experiment; a thicker SWB results in a larger the GPR detection error.

## Discussion

1 Unlike the numerically modeled simulation (Fig 5), a greater level of clutter is observed within the SWB on radar-grams from the laboratory experiment; this phenomenon may be caused by an uneven moisture distribution within the SWB. To verify this speculation, we measured the moisture contents of different SWB layers, and the results are shown in Table 4.

Table 4 shows a slightly decreasing trend in the moisture content within different SWB layers with different irrigation volumes from the upper to lower layers. This reduction in moisture would lead to very slight reductions in the radar wave velocity. This difference in velocity, if abrupt, might result in some clutter, which would increase the difficulty of interpreting the radar-gram. This phenomenon is consistent with the study by Klenk et al. [33], who showed that the transition zone above a ground water table in a homogeneous medium displays a smooth variation in dielectric permittivity, and this permittivity variation produces a corresponding GPR reflection resulting from the coherent superposition of an infinitesimal contribution along a transition zone or capillary fringe.

2 GPR detection errors for SWBs with different irrigation volumes show that the accuracy of the thickness detection is related to the SWB thickness. A greater thickness results in a lower detection accuracy, which may be related to the speed dispersion of electromagnetic waves. A thicker SWB results in a greater speed of dispersion and subsequently, a greater GPR detection error of the thickness. Similar effects have been observed in soil moisture and water table studies. Bano [5] theoretically investigated the reflection of GPR waves from the transition zone above a water table and found that the saturation degree of the capillary fringe gradually decreases upward, implying an increase in the velocity of the GPR waves. According to Redman et al. [34], the GPR-measured water content is substantially influenced by variations in the water content distribution, and the major source of error is believed to be related to surface scattering and spatial variability within the GPR sampling volume.

**Table 4:** Stratified volume moisture content of the SWB.

Irrigation volume (ml)	Upper layer (0–3 cm)	Middle layer (3–6 cm)	Bottom layer (6–9 cm)
200	0.197	0.193	0.156
400	0.163	0.15	0.142
600	0.154	0.155	0.119

## Conclusions

In this paper, a method that combines a numerical simulation, laboratory experiment and field measurements is used to verify the nondestructive detection of SWB with GPR. The following conclusions were drawn:

1. The results of the numerical simulation, soil laboratory experiment, soil field experiment and excavation profiles indicate that GPR detects the position and thickness of the SWB based on observable reflection characteristics on the original GPR radar-gram. The upper interface of the SWB is a coherent reflector with a length that is proportional to the surface diameter of the SWB. The bottom interface of the SWB is a hyperbola facing in the downward direction, where the vertex is the location of the bottom of the SWB.
2. Although the measured SWB results are consistent with the numerical simulation, the discrimination of SWBs will be different under different conditions. In the numerical simulation images, essentially no clutter was observed, and the hyperbolas formed by the reflections from SWB bottom interfaces were clear and distinct. In contrast, clutter and background noise were present and interfered with the interpretation of both laboratory and field radar-grams. Particularly in the field detection environment, only the SWBs with larger dimension scans were clearly identified using GPR, which is different from the laboratory experiment.
3. The average GPR detection error for the SWB thickness was low, which confirms the effectiveness and feasibility of using GPR to detect SWBs. Based on these results, the detection accuracies will be related to the thickness of SWBs. In general, thicker SWBs may be more observable but will have lower detection accuracies than shallow SWBs. The effective detection of SWBs with GPR also depends on soils and field conditions.
4. The F-K migration process effectively converges and gathers the SWB diffraction wave, and suppresses the interference from multiple waves to a certain extent; as a result, the clarity of some GPR images is improved. The shape of the SWB in the image after the F-K offset migration processing is similar to the excavation profile. Therefore, F-K offset migration processing for the GPR radar-gram replicates the SWB shape, which makes the GPR interpretation of the SWB more intuitive and effectively reduces difficulties in the interpretation of some GPR radar-grams.

The experiments also have some limitations. In the field experiments, because the soil media was wet and contained some scattered crop stalks and roots, the intact SWB was not successfully excavated. We are attempting to find an ideal solution for this problem.

## Supporting information

S1 Raw image Original image. (DOCX)
