# Supplementary figures and images for: The application of GPR to the detection of soil wetted bodies formed by drip irrigation
Source: PLoS One. 2020 Jul 22;15(7):e0235489. doi: 10.1371/journal.pone.0235489 (PMC7375588; doi:10.1371/journal.pone.0235489)

# Original image


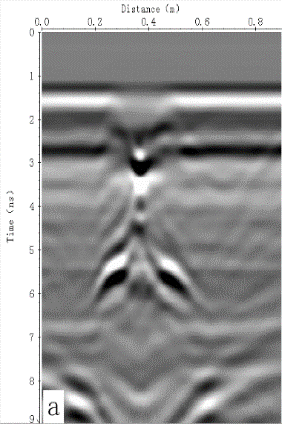

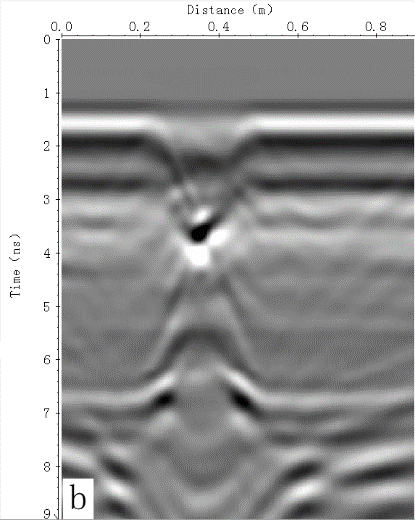

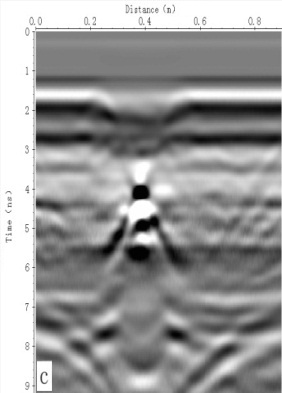


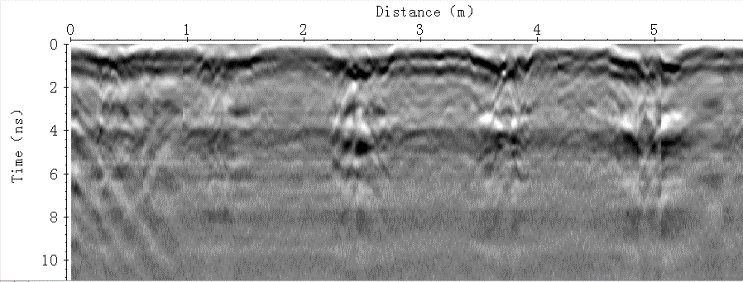

Supplement: S1 Raw image — (DOCX) [file pone.0235489.s001.docx]
